# Identification of risk factors of severe hypersensitivity reactions in general anaesthesia

**DOI:** 10.1186/s12948-015-0017-9

**Published:** 2015-06-22

**Authors:** Corrado Mirone, Donatella Preziosi, Ambra Mascheri, Gianluigi Micarelli, Laura Farioli, Luca G Balossi, Joseph Scibilia, Jan Schroeder, Laura M Losappio, Maria G Aversano, Chrysi Stafylaraki, Michele Nichelatti, Elide A Pastorello

**Affiliations:** Allergology and Immunology Unit, Niguarda Ca’ Granda Hospital, Milan, Italy; Department of Laboratory Medicine Niguarda Ca’ Granda Hospital, Milan, Italy; Service of Biostatistics, Niguarda Ca’ Granda Hospital, Milan, Italy; Allergology and Immunology Unit, Niguarda Ca’ Granda Hospital and Department of Clinical Science and Community Health Università degli Studi of Milan, Milan, 20162 Italy

**Keywords:** General anaesthesia, Hypersensitivity, Risk factors, Anaphylaxis, Severity, Neuromuscular blocking agents, Serum tryptase, Age, Hypertension, Angiotensin-converting enzyme inhibitor

## Abstract

**Background:**

Hypersensitivity reactions to anaesthetic agents are rare but often severe, with a mortality ranging from 4 to 9% in IgE-mediated events. Identification of the risk factors may contribute to limit the incidence of these reactions. The aim of our study was to search for possible risk factors of severe perioperative hypersensitivity reactions in our study population.

**Methods:**

For this study we retrospectively reviewed data from 193 patients who experienced drug hypersensitivity reactions during general anaesthesia. The diagnostic protocol consisted of 1) history of the reaction, 2) measurement of serum baseline tryptase and specific IgE-assays for latex, beta-lactams and succinylcholine, 3) skin tests for the agents listed in the anaesthesia chart and for others likely to be safe for future use, latex, and others medications administered during the perioperative period (i.e. antibiotics), 4) subdivision of our patients on the basis of two criteria: a) grade of severity of clinical reactions according to the Ring and Messmer classification; b) results of skin tests and/or serum specific IgE-assays.

**Results:**

One hundred of 193 patients had reactions of grade I, 32/193 patients had reactions of grade II, 55/193 patients had reactions of grade III and 6/193 patients had reactions of grade IV. A diagnosis of IgE-mediated reaction was established in 55 cases (28.50%); the most common causes were neuromuscular blocking agents, followed by latex and beta-lactams. Severe reactions were associated with older age (p = 0.025), asthma (p = 0.042), history of hypertension (p = 0.001), intake of serum angiotensin converting enzyme inhibitor medication (p = 0.012) or serum angiotensin II antagonist (p = 0.033), higher levels of basal tryptase (p = 0.0211). Cardiovascular symptoms (p = 0.006) and history of hypersensitivity to antibiotics (p = 0.029) were more frequently reported in IgE-mediated reactions.

**Conclusions:**

We confirmed the relevance of several clinical features as risk factors for anaphylactic reactions induced by anaesthetic agents: older age, asthma, hypertension and antihypertensive drugs. We observed increased levels of serum basal tryptase in severe reactions: this finding may signify that this biomarker is useful for the identification of patients at risk.

**Electronic supplementary material:**

The online version of this article (doi:10.1186/s12948-015-0017-9) contains supplementary material, which is available to authorized users.

## Background

Severe hypersensitivity reactions observed during general anaesthesia are among the most frequent forms of drug-induced anaphylactic reactions as recently shown in studies from Australia [1], UK [2] and USA [3]. The incidence of immediate allergic reactions during general anaesthesia has been reported between 1 in 20000 and 1 in 10000, with a mortality rate ranging from 3 to 9%, depending on the country [4]. In an Australian study [1], the main cause of 112 anaphylaxis fatalities was drug allergy: this was the only trigger showing a definite increase in the observation period. When the culprit drug was specified, anaesthetic agents were identified as the second most common cause in 64 deaths attributable to medication (5/64) [1]. In a French survey [5] conducted between 1997 and 2004, 2516 reactions were reported, with an estimated incidence of IgE-mediated reactions of 100/million procedures and a female predominance in adults. The distinction between allergic and non-allergic forms was made on the basis of diagnostic tests [6]. Clinical manifestations were more severe in IgE-mediated than in non–IgE-mediated reactions. Neuromuscular blocking agents (NMBAs) were the most common cause of these events. General risk factors identified for drug-induced anaphylaxis fatalities [1] were age 55 to 85 years, the coexistence of cardiovascular or respiratory diseases and the exposure to antibiotics or anaestethic agents. The only risk factor specifically identified for immediate perioperative anaphylactic reactions was a previous history of adverse reaction after exposure to general anaesthetics; there was no association between IgE mediated reactions and asthma, atopy, drug hypersensitivity in general or previous exposure to anaesthetics [5]. On the contrary, allergic reactions to Hymenoptera stings, angiotensin converting enzyme (ACE) inhibitors, male sex, serum tryptase levels above 5 ng/mL [7] and systemic mastocytosis [8] were identified as risk factors of anaphylaxis. In food allergy an association between type and severity of pre-existing allergic disease and clinical features of anaphylaxis was also observed: an obstruction of the upper airways was more frequent in patients with non-controlled allergic rhinitis, while the lower airways were often affected in the presence of severe extrinsic asthma; cardiovascular system was involved mostly in patients with severe and widespread eczema [9]. Asthma was considered a major risk factor for fatal food-induced anaphylaxis [10,11]. It has been reported that all anaphylactic reactions induced from any trigger occurred more often and more severely in patients affected by non-controlled allergic rhinitis, eczema, and to a greater extent by asthma, COPD [9,12] and other respiratory diseases, cardiovascular diseases [13], systemic mastocytosis and/or other mast cell-related disorders [14-16] and on therapy with ACE-inhibitors [17]. The purpose of our study was to identify predisposing factors to perioperative anaphylaxis. To this aim we associated several clinical and laboratory parameters with the grade of severity of documented hypersensitivity reactions occurring during surgical procedures in a population of patients referred to our center.

## Methods

### Patients

#### Patients with perioperative hypersensitivity reactions

We collected retrospective data from all of the patients with a history of hypersensitivity perioperative reactions referred to our allergy clinic during the period January 1, 2010 - May 31, 2014. We adopted a standardised protocol including detailed clinical history of the reaction, skin tests (skin prick test and intradermal testing), in vitro tests when available (i.e. specific serum IgE to latex, succinylcholine and beta-lactams). In 66 out of 193 patients basal serum tryptase levels were determined. Skin testing was performed according to published guidelines [4,18]. All of the patients were enrolled after signing an informed consent to the collection of their clinical and laboratory data. For all the selected patients, the association between the risk of severe hypersensitivity reactions in general anaesthesia and age, asthma and other respiratory diseases, antihypertensive medication, basal serum tryptase, gender, atopy, history of reactions to non-steroidal anti-inflammatory drugs (NSAIDs) or antibiotics, allergic contact dermatitis, urticaria, thyroid diseases and diagnosis of IgE-mediated reactions to general anaesthetics was evaluated by a statistical analysis.

### In vivo tests

#### Skin tests procedures

All of the patients underwent skin tests for the drugs listed in the anaesthetic record, as well as for latex and other medications or products administered during the perianesthetic period [4], when available.

##### Anaesthetic drugs

Immediate hypersensitivity reactions to anaesthetic drugs were investigated using skin prick tests (SPTs) and intradermal tests (IDTs) with commercial solutions that were performed according to the recommendations of the *Société Française d’Anesthésie et de Réanimation* and the *Société Française d’Allergologie* approved by the members of the European Network for Drug Allergy (ENDA) [4]*.* Briefly, drugs were tested on the volar forearm at the concentrations normally unable to induce localized skin irritation, to minimize the false-positive results. We used 0.9% saline control as negative control and histamine 10 mg/mL as a positive control. SPTs were read after 20 minutes and were considered positive when a wheal diameter at least half the diameter of the positive control or 3 mm greater than the negative control was seen. IDTs were undertaken when SPTs were negative. A bleb of up to 4 mm in diameter was produced injecting into the dermis 0.02 to 0.05 mL of the drug at different dilutions. A positive result consisted in a persistent, often pruritic*,* erythematous wheal after 20 minutes, the diameter of which was at least twice that of the post-injection bleb. The anaesthetics agents were tested at the concentrations listed in Table 1 [4].

**Table 1:** **Concentrations of anaesthetic agents tested**

**Available agents**	**Skin Prick Test**		**Intradermal Tests**		
	mg/mL	Dilution	mg/mL	Dilution	mcg/mL
Atracurium	10	1/10	1	1/1000	10
Cisatracurium	2	Undiluted	2	1/100	20
Mivacurium	2	1/10	0.2	1/1000	2
Pancuronium^*^	2	Undiluted	2	1/10	200
Rocuronium	10	Undiluted	10	1/200	50
Succinylcholine	50	Undiluted	50	1/500	100
Vecuronium	4	Undiluted	4	1/10	400
Midazolam	5	Undiluted	5	1/10	500
Propofol	10	Undiluted	10	1/10	1000
Thiopental	25	Undiluted	25	1/10	2500
Ketamine	10	1/10	10	1/10	1000
Fentanyl	0.05	Undiluted	0.05	1/10	5

(^*^) untested because unavailable since 2012

##### Beta-lactam drugs

Investigation for beta-lactams allergy was performed as reported in the ENDA position statement [19].

##### Latex

SPT for latex was performed using standardized commercial natural rubber latex extract from two manufacturers (Alk Abello®, Hørsholm, Denmark; Stallergènes®, Antony, Hauts-de-Seine, France) as reported above for anaesthetic drugs.

### In vitro tests

#### Tryptase

Serum basal tryptase (sBT) was measured with an immunofluorimetric assay by the ImmunoCAP System (Phadia, now Thermo Fisher Scientific, Uppsala, Sweden). A measurement of tryptase level was performed for all of the patients during an asymptomatic period, at least 6 weeks after the last anaphylactic/systemic allergic event. The cutoff value of tryptase was considered to be ≥5 ng/mL, according to the in house reference obtained from a sample of hundreds of healthy volunteer subjects resident in Northern Italy as recommended by manufacturer.

#### Serum specific IgE

The presence of specific IgE antibodies to latex, succinylcholine, penicilloyl V, G, ampicilloyl, amoxicilloyl and cefaclor (normal value ≤ 0.10 IgE kUA/L) were determined using the ImmunoCAP System (Phadia, now Thermo Fisher Scientific, Uppsala, Sweden) in accordance with the manufacturer’s instructions.

### Diagnosis and severity grading

In this retrospective analysis, immediate IgE-mediated hypersensitivity reactions were diagnosed according with skin tests and/or IgE assay results consistent with the clinical history [20]. The severity of reactions was graded retrospectively using the four classes scale adapted from Ring and Messmer [21].

### Statistical analysis

All of the collected variables were analysed with the usual descriptive methods: continuous variables were described by mean and standard deviation or by median and range, based on their distribution (checked by visual inspection of the histogram and – in the case – also by the Shapiro-Wilk test), while categorical variables were described by relative and absolute frequency tables. Cross-tabulations between categorical variables were analysed by the Fisher’s exact test. The differences of continuous variables with respect to the reaction’s severity were analysed by two-sided Student’s *t* test (in the case, with the Welch’s correction for different variances), or Mann–Whitney U test. The one-way ANOVA, followed (if case of significant F test) by Sidak pairwise comparisons was used to evaluate the differences between continuous variables among more than two groups.

The continuous variables of interest, as well as the possible confounders such as age and sex, were then fitted as independent regressors in a set of logistic models, using the Wald’s test to verify the significance of each regressor, as well as the likelihood ratio test to check the significance of the model as a whole; in these logistic regressions the reaction severity was taken into account as dependent binary variable. To obtain reliable cutoffs of some continuous variables, the receiver operating characteristics (ROC) curve analysis was carried out, and the Youden’s method was used to choose for the optimal cutpoints.

Statistical significance was assumed for p < 0.05; all calculations were carried out using the Stata/SE 13.1 statistical package.

## Results

### Patients

A total of 193 patients (71 males and 122 females, mean age 37.5 ± 20.8 years) with peri-operative hypersensitivity reactions during general anaesthesia were included in this retrospective observational cohort study. A summary of patients’ demographic and clinical data are shown in Table 2. A total of 100/193 patients (51.81%; 39 males and 61 females) had reactions of grade I, 32/193 patients (16.58%; 12 males and 20 females) had grade II reactions, 55/193 patients (28.49%; 20 males and 35 females) had grade III reactions, and 6/193 patients (3.10%; only females) had grade IV reactions. For the statistical analysis, the patients were further classified into two groups: group 0 consisting of 132/193 patients (68.40%) (mean age: 35.16 ± 19.58 years) with grade I and II and group 1 consisting of 61/193 patients (31.60%) (mean age: 42.56 ± 22.64 years) with grade III and IV reactions. NMBAs skin tests were performed in 130/193 patients (67.35%, 47 males and 83 females, mean age 38.95 ± 20.49 years), and they were positive in 27/130 patients (20.76%). In each of the 130 cases, the severity group of the reaction was 0 (grade I + II) in 85/130 patients (65.38%) and 1 (grade III + IV) in 45/130 patients (34.61%). The respective contribution of each neuromuscular blocking drug is shown in Table 3. We carried out skin tests with five different NMBAs, as cross-reactions occurs commonly among these drugs, in order to identify a safe alternative agent for subsequent anaesthesia. We did not report our cross-reactivity results because of the small number of positive skin tests to NMBA in our patients. Serum measurements of succinylcholine specific IgE levels were performed in 14/16 patients (87.5%) who were exposed to this agent during general anaesthesia; all of the patients were negative. Skin tests with other anaesthetic agents, as reported in Table 4, were positive in 3 patients for ketamine, and in 1 patient for atropine. SPTs to latex were positive in 6/193 patients (3.10%). Serum specific latex-IgE assay was positive in 16/68 patients (23.52%). Investigations for beta-lactams allergy were positive in 11/89 patients (12.35%).

**Table 2:** **Demographic and clinical characteristics of patients with a history of reaction during general anaesthesia**

	**n**	**%**
**No. of patients**	193	
**Age (y), mean (range)**	37.5 (2–84)	
**Sex (F/M)**	122/71	
**Personal history of allergic disease**	84	43.52
**Severity of reaction**		
Grade I (F/M)	100 (61/39)	51.81
Grade II (F/M)	32 (20/12)	16.58
Grade III (F/M)	55 (35/20)	28.49
Grade IV (F/M)	6 (6/0)	3.10
**Concomitant diseases**		
Asthma	37	19.17
Other respiratory diseases	10	5.18
Hypertension	30	15.54
Thyroid diseases	13	6.73
Chronic urticaria	13	6.73
**Anti-hypertensive agents**		
ACE-inhibitors	14	7.25
ARBs	9	4.66
Beta-blockers	13	6.73

No.: number, ACE: angiotensin converting enzyme, ARBs: angiotensin II receptor blockers

**Table 3:** **Positivity to NMBAs skin tests**

**NMBAs**	**Patients exposed during anaesthesia (F/M)**	**Positivity to skin test in patients exposed (F/M)**
*Vecuronium*	22 (13/9)	4 (3/1)
*Atracurium*	18 (12/6)	3 (2/1)
*Cisatracurium*	23 (13/10)	1 (1/0)
*Rocuronium*	19 (15/4)	1 (1/0)
*Mivacurium*	8 (4/4)	0
*Pancuronium*	3 (2/1)	0
*Succinylcholine*	15 (11/4)	0

NMBAs: Neuromuscular Blocking Agents, F: female, M: male

**Table 4:** **Skin test results to agents other than NMBAs involved in hypersensitivity reactions during anaesthesia**

**Drugs**	**Positive skin tests (%)**	**No. of patients tested**
**Beta-lactams**	4 (8.16)	49
*Penicillin*	1 (2.04)	
*Cephalosporin*	3 (6.12)	
**Hypnotics**		8
*Ketamine*	3 (37.50)	
**Others**		87
*Atropine*	1 (1.14)	
**Latex**	6 (3.10)	193

NMBAs: Neuromuscular Blocking Agents

#### Allergic diagnostic work-up

At the end of the allergy work-up, a diagnosis of IgE-mediated hypersensitivity reaction was established in 55 cases (28.50%), the remaining 138 cases (71.50%) were considered as non-IgE-mediated reactions. The different clinical symptoms of IgE- and non IgE-mediated reactions observed during general anaesthesia are reported in Table 5. In our group clinical expression was not more severe in patients with a documented IgE-mediated reactions to general anaesthetics (OR: 0.906, CI95%: 0.519 to 1.581; Wald’s test: p = 0.729). However, cardiovascular symptoms (bradycardia, hypotension, cardiovascular collapse, cardiac arrest) reported in 29/193 patients (15.03%), were more frequent in IgE-mediated reactions (27.27% vs 10.14%; Fisher’s exact test: p = 0.006). No significant difference was observed between IgE- and non-IgE-mediated reactions as regards mucocutaneous symptoms (erythema, or urticaria, or angioedema) (67.27% vs 74.63%; Fisher’s exact: p = 0.372). Bronchospasm was recorded more often in patients affected by asthma (30.65% vs. 13.74%; Fisher’s exact test: p = 0.010), but not in IgE-mediated reactions in particular (38.18% vs. 29.71%; Fisher’s exact test: p = 0.306).

**Table 5:** **Patients’ distribution according to clinical symptoms and to mechanism of hypersensitivity reactions during anaesthesia**

**Symptoms group**	**IgE-mediated hypersensitivity**		**No. of patients**	**P value**
**Mucocutaneous**	**0**	**1**		
**0**	35 (25.36%)	18 (32.72%)	53	0.372
**1**	103 (74.63%)	37 (67.27%)	140	
**Cardiovascular**	**0**	**1**		
**0**	124 (89.86%)	40 (72.73%)	164	0.006
**1**	14 (10.14%)	15 (27.27%)	29	
**Bronchospasm**	**0**	**1**		
**0**	97 (70.29%)	34 (61.82%)	131	0.010
**1**	41 (29.71%)	21 (38.18%)	62	
**No. of patients**	**138**	**55**	**193**	

0 = absent 1 = present

Mucocutaneous: erythema and/or urticarial and/or angioedema

Cardiovascular: hypotension and/or cardiovascular collapse and/or cardiac arrest

History of hypersensitivity to antibiotics (OR: 2.024, CI95%: 1.076 to 3.807; Wald’s test: p = 0.029) was more common in IgE- than in non IgE-mediated reactions.

No significant differences were observed between the two groups (IgE- and non-IgE-mediated reactions) as regards age (OR: 0.999, CI95%: 0.984 to 1.014; Wald’s test: p = 0.912), sex (OR: 0.682, CI95%: 0.363 to 1.280; Wald’s test: p = 0.234), atopy (OR: 0.956, CI95%: 0.776 to 1.177; Wald’s test: p = 0.675), asthma (OR: 0.701, CI95%: 0.308 to 1.599; Wald’s test: p = 0.400) and other respiratory diseases (OR: 0.989, CI95%: 0.246 to 3.969; Wald’s test: p = 0.988), allergic contact dermatitis (OR: 0.511, CI95%: 0.140 to 1.869; Wald’s test: p = 0.311), urticaria (OR: 2.093, CI95%: 0.671 to 6.527; Wald’s test: p = 0.203), therapy with beta-blockers (OR: 1.485, CI95%: 0.464 to 4,751; Wald’s test: p = 0.504), ACE-inhibitors (OR: 1.817, CI95%: 0.600 to 5.496; Wald’s test: p = 0.290) or ARBs (OR: 1.154, CI95%: 0.278 to 4.783; Wald’s test: p = 0.843), thyroid diseases (OR: 1.485, CI95%: 0.464 to 4.751; Wald’s test: p = 0.504), serum tryptase values at baseline (measured in 66/193 patients) (OR: 0.830, CI95%: 0.648 to 1.064; Wald’s test: p = 0.143), and previous adverse reactions to NSAIDs (OR: 1.164, CI95%: 0.579 to 2.341; Wald’s test: p = 0.669).

#### Risk factors

**Figure 1:**
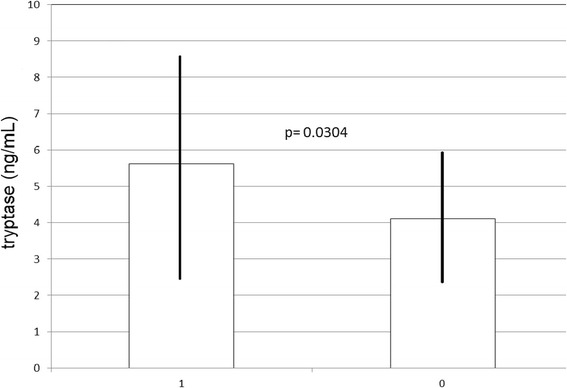
**Basal serum tryptase levels and hypersensitivity reaction severity in general anaesthesia:** Mean basal serum tryptase values ± standard deviation between group 1 (severity grade III + IV) and group 0 (severity grade I + II).

We looked for risk factors of severe allergic reactions (i.e. grade III and IV) during the perioperative period:

1. **Age.** Each additional year of age increased the risk of severe reaction by 1.7% (OR: 1.017, CI95% :1.002 to 1.032; Wald’s test: p = 0.025)
2. **Asthma and antihypertensive medication**. A significant association was observed between the development of a severe reaction during the general anaesthesia and history of asthma (OR: 2.144, CI95%: 1.028 to 4.471; Wald’s test: p = 0.042) and ongoing treatment with ACE inhibitors (OR: 4.326, CI95%: 1.383 to 13.530; Wald’s test: p = 0.012) or ARBs (OR: 4.703, CI95%: 1.134 to 19.498; Wald’s test: p = 0.033). Hypertension was significantly more frequent in patients of group 1 (severity grade III + IV) than in patients of group 0 (severity grade I + II) (29.51% vs. 9; Fisher’s exact test: p = 0.001).
3. **Basal tryptase:** sBT tryptase levels were measured in 66/193 patients: values ranged from 1.5 to 14.5 ng/mL (mean 4.73 ± 2.54 μg/L); 39/66 patients (59.09%) belonged to group 0 (severity grade I + II), and 27/66 patients (40.91%) to group 1 (severity grade III + IV). No patient had sBT levels above 20 ng/ml, a minor diagnostic criteria for mastocytosis. There was a significant association between sBT and hypertension: any unitary increase of sBT levels resulted in an increase in the odds of suffering from hypertension by 30% (OR:1.296, CI95%: 1.023 to 1.643; Wald’s test: p = 0.032). In addition, when we randomly extracted two subjects, one with hypertension, and one without, we found a 74.2% probability that the first patient showed higher sBT concentrations (Mann–Whitney U test: p = 0.004). Furthermore, by one-way ANOVA we found that sBT values increased significantly in the grade of severity of reactions from grade I to grade III (F test: p = 0.021), with Sidak pairwise test respectively giving p = 0.140 (I vs. II), p = 0.694 (I vs. III) and p = 0.017 (II vs. III). Grade IV was not considered in this analysis because of the limited number of patients. This finding was confirmed from the observation that sBT values were significantly higher in patients of group 1 (severity grade III + IV) (mean: 5.62 ± 3.14; range: 1.5-14.5) than in patients of group 0 (severity grade I + II) (mean: 4.11 ± 1.83; range: 1.5-9.4) (Welch’s test: p = 0.0304) (Figure 1). In this subgroup of patients age did not affect the severity of the reaction (OR: 1.023; CI95%: 0.996 to 1.050; Wald’s test: p = 0.102).

No effect on symptom severity was observed as regards sex (OR: 1.306, CI95%: 0.689 to 2.477, Wald’s test: p = 0.412), atopy (OR: 0.949, CI95%: 0.762 to 1.181; Wald’s test: p = 0.640), history of hypersensitivity to NSAIDs (OR: 0.777; CI95%: 0.381 to 1.584; Wald’s test: p = 0.489) or antibiotics (OR: 0.984; CI95%: 0.533 to 1.816; Wald’s test: p = 0.960), allergic contact dermatitis (OR: 0.974, CI95%: 0.323 to 2.936; Wald’s test: p = 0.963), other respiratory diseases (OR: 3.43, CI95%: 0.932 to 12.662; Wald’s test: p = 0.064), urticaria (OR: 0.943, CI95%: 0.278 to 3.193; Wald’s test: p = 0.925), therapy with beta-blockers (OR: 1.916, CI95%: 0.615 to 5.968; Wald’s test: p = 0.262), thyroid diseases (OR: 1.361, CI95%: 0426 to 4.348; Wald’s test: p = 0.602). Furthermore, we did not find a significant association between sensitization to NMBAs and the following variables: sex (15.49% vs 13.11%; Fisher’s exact test: p = 0.671), higher grade of severity of the reaction (11,48% vs 15,15%; Fisher’s exact test: p = 0.656), asthma (10.81% vs 14.74%; Fisher’s exact test: p = 0.792), history of hypersensitivity to NSAIDs and/or antibiotics (16.07% vs 11.11%; Fisher’s exact test: p = 0.403), atopy (11.90% vs 14.95%; Fisher’s exact test: p = 0.672), age (OR: 0.989, CI95%: 0.969 to 1.009; Wald’s test: p = 0.308), therapy with ACE-inhibitors (14.29% vs 13.97%; Fisher’s exact test: p > 0.999), ARBs (0% vs 14.67%; Fisher’s exact test: p = 0.365), or beta-blockers (15.38% vs 13.89%; Fisher’s exact test: p > 0.999), allergic contact dermatitis (3.70% vs 96.30%; Fisher’s exact test: p = 0.704). We observed a nearly significant association between allergy to latex and history of food allergy (14.29% vs 4.29%; Fisher’s exact test: p = 0.059).

## Discussion

In our study we looked for risk factors of severe adverse reactions in patients with history of perioperative hypersensitivity. A main finding of our study was that older patients were confirmed to be affected by more severe hypersensitivity reactions during general anaesthesia in comparison with younger subjects. Previous studies identified older patients as being at risk of severe and fatal anaphylaxis, in particular for reactions induced by drugs and Hymenoptera venom [2,22,1,13,7,23,24,3]. The higher risk of severe reaction or demise with respect to increasing age has already been reported [13,23,24] and has been attributed to cerebrovascular or cardiovascular diseases, capable of reducing the patients’ ability to tolerate some complications of anaphylaxis (i.e. hypotension, hypoxia and arrhythmia) or the adverse effects of the treatment in the presence of these conditions. An alternative explanation was the exposure to consecutive triggers such as drugs [24]. A second important finding was a significant association between previous respiratory and cardiovascular diseases as asthma and hypertension and the severity of perioperative reactions as previously reported in literature in different allergic conditions [2,13,24]. Bronchospasm has been a major component of fatal reactions in children and of most fatalities due to food allergy [2] and asthma was more common in patient with food related anaphylaxis [3]. The significance of cardiovascular diseases as risk factors for severe clinical manifestation was also consistent with the observation reported above i.e. older adults may be at greater risk of exposure to medications triggering anaphylactic reactions. We pointed out hypertension as a risk factor independent from the use of antihypertensive drugs. However ACE inhibitors and ARBs were also significantly associated with the risk of anaphylaxis during general anaesthesia; the role of these drugs as facilitating factors of severe anaphylaxis has already been discussed by other authors especially as regards ACE inhibitors [7,17,24,25]. To our knowledge, this is the first investigation evaluating the behavior of sBT as a predictor of severe reactions in patients with perioperative hypersensitivity. We found that higher levels of sBT were associated with more severe clinical symptoms in IgE- and non IgE-mediated reactions observed during general anaesthesia. Furthermore our data set on sBT have shown that a value > 4.9 ng/mL gives a sensitivity and a positive predictive value (PPV) of 59.3% in hypersensitivity during general anaesthesia, but more studied are needed to improve the cutoff value. Nevertheless this result confirmed previous findings in anaphylaxis induced by different agents, as Hymenoptera venom [7], in which a sBT value of about 5 ng/mL clearly increased the odds ratio of severe systemic reactions. We found a significant association between sBT values and hypertension: a possible explanation was an increase of sBT in cardiovascular diseases [26,27]. We found that the association of sBT level with the severity of reactions was not influenced by patients’ age in spite of the previously described increase of serum levels of this mediator in older subjects [28,29]. Until now serum tryptase has proved useful as a diagnostic tool of anaphylactic events during general anaesthesia during the acute phase with a value >25 ng/mL [4,30]. This threshold was associated with a definite increase of total tryptase in the acute event and not with a false positive value due to unapparent mastocytosis. In our study we observed for the first time that sBT can be used as a screening tool to identify patients at risk of anaphylaxis in the perioperative period. We did not confirm an association between IgE-mediated reactions and the severity of all symptoms reported from our patients as found in other studies [5,30]. Nonetheless, we found that cardiovascular symptoms, the index of the most severe reactions according to Ring and Messmer classification, were significantly more frequent in patients with IgE-mediated reactions.

## Conclusions

In summary our study confirmed the relevance of several clinical features as risk factors of anaphylactic reactions induced by anaesthetic agents: older age, asthma, hypertension and antihypertensive drugs. We observed increased levels of serum basal tryptase in severe reactions: this finding may signify that this biomarker is useful for the identification of patients at risk.

## Abbreviations

NMBAs: Neuromuscular blocking agents
ACE: Angiotensin converting enzyme
NSAIDs: Non-steroidal anti-inflammatory drugs
SPT: Skin prick test
IDT: Intradermal test
ENDA: European Network for Drug Allergy
sBT: Serum basal tryptase
